# Revisiting Left Atrial Appendage Closure Versus Oral Anticoagulants in Recurrent Atrial Fibrillation Management: An Updated Systematic Review and Meta-Analysis

**DOI:** 10.7759/cureus.70854

**Published:** 2024-10-04

**Authors:** Guilherme Requião Radel Neto, Lucas Bittar de Morais, Raphael Hideki Sheguti, André Poci Liberato

**Affiliations:** 1 Medicine Department, Anhembi Morumbi University, São Paulo, BRA; 2 Endovascular Surgery and Angiography Department, Regional Hospital of Sorocaba "Dr. Adib Domingos Jatene", Sorocaba, BRA

**Keywords:** atrial fibrillation, direct oral anticoagulants, left atrial appendage closure, meta-analysis, stroke

## Abstract

Atrial fibrillation (AF) is a significant public health problem due to its association with coronary heart disease, stroke, and mortality, especially in the elderly. Therefore, traditional warfarin therapy, direct oral anticoagulants (DOACs), and the recent left atrial appendage closure (LAAC) have been compared as treatment approaches. In this regard, we aimed to synthesize the current evidence regarding the comparison these mentioned modalities in patients with AF. A comprehensive database search for records comparing LAAC and DOACs in patients with AF was conducted until December 15, 2023. An updated meta-analysis was conducted using fixed and random effect models to calculate odds ratios (OR) or mean differences (MD) with 95% confidence intervals (CIs) for dichotomous and continuous outcomes, respectively. Eleven studies were eligible that included a total of 68171 patients. Compared to DOACs, the LAAC group had a lower rate of hospital stay duration (MD -1.23; 95% CI -1.51 to -0.95; *p* < 0.001). There was no statistically significant difference between LAAC and DOACs in terms of the composite outcome of stroke, systemic embolism, cardiovascular death, all-cause mortality, ischemic stroke and thromboembolic events ischemic, major bleeding and cardiovascular mortality (OR 0.83, 95% CI 0.27-2.48, *P *= 0.73). Our meta-analysis showed a lower rate of hospital stay duration that favors LAAC. However, the risk of composite outcomes of stroke, systemic embolism, cardiovascular death, all-cause mortality, ischemic stroke, thromboembolic events, ischemic stroke, major bleeding, and cardiovascular mortality was similar between the two treatment groups.

## Introduction and background

Atrial fibrillation (AF) is the most common sustained cardiac arrhythmia associated with coronary heart disease, stroke, and mortality, affecting more than 33 million patients worldwide [1,2]. AF has increased almost 1.1-fold in incidence and 1.4-fold in mortality between 1990 and 2019 [2]. Furthermore, patients with AF have a five-fold risk of developing stroke or systemic embolism, with approximately 25% of all strokes occurring in the elderly. Thus, prevention of stroke and thromboembolic events is the mainstay in managing patients with AF [3]. Moreover, vitamin K antagonists such as warfarin were traditionally the mainstay therapy in these patients. However, several limitations of warfarin (coumadin) have shed light on other treatment options, including a narrow therapeutic window, interaction with other drugs or different types of food, constant monitoring, and high bleeding tendency [4,5]. Direct oral anticoagulants (DOACs) have been developed to overcome these limitations and have proven to be non-inferior and even superior to warfarin [3]. DOACs (e.g., dabigatran, rivaroxaban, apixaban, and edoxaban) are now the standard of care for the prevention of stroke and thromboembolic events in patients with AF [5].

In contrast, DOACs also have some limitations, especially in elderly patients with AF, such as a history of bleeding, or in AF patients who are intolerant and/or contraindicated to long-term oral anticoagulation therapy [3,6]. Therefore, given that the left atrial appendage produces up to 90% of the responsible thrombi, percutaneous left atrial appendage closure (LAAC) has been recently considered as an alternative to warfarin in patients with AF, with proven non-inferiority to warfarin in the literature [5]. LAAC involves percutaneous placement of a device to occlude the left atrial appendage (LAA), thereby preventing thrombus formation and reducing the need for long-term anticoagulation. Although the PLAATO device (eV3, Inc., Plymouth, MA) was the first device to be implanted in humans, it is not commercially available. Watchman (Boston Scientific, Natick, MA) and Amplatzer Cardiac Plug (St. Jude Medical, Minneapolis, MN) are now the two main available devices [7].

Only one randomized controlled trial (RCT) and multiple observational studies have evaluated the safety and efficacy of LAAC and DOACs in patients with AF. However, there is still uncertainty regarding the optimal strategy for preventing stroke and thromboembolic events in patients with AF. By synthesizing and summarizing the current evidence in an updated systematic review and meta-analysis, we aimed to provide an up-to-date comparison of the safety and efficacy of LAACs and DOACs in patients with AF.

This article was presented as an abstract at the 2024 Brazilian Society of Cardiology Annual Scientific Meeting on September 21, 2024, in an e-poster format. The full published work will be made available through Cureus. This study has a Protocol Registration of CRD42024496719 (PROSPERO).

## Review

Materials and methods

We followed the Preferred Reporting Items for Systematic Reviews and Meta-Analyses (PRISMA) statement guidelines for reporting this updated systematic review and meta-analysis [8]. All steps were performed in strict accordance with the Cochrane Handbook of Systematic Reviews and Meta-analysis of Interventions (version 5.1.0) [9]. We compared the safety and efficacy of LAAC and DOACs in patients with AF in terms of the composite outcomes of stroke, systemic embolism, cardiovascular death, all-cause mortality, ischemic stroke, thromboembolic events, ischemic stroke, major bleeding, cardiovascular mortality, and hospital stay duration.

Eligibility Criteria

The inclusion criteria involved all RCTs, propensity score-matched studies, and cohort studies comparing LAAC with DOACs in patients with AF with available data in both arms on the targeted outcomes with no limitations on the year of publication. The exclusion criteria included studies published in languages other than English, case series or reports, any observational studies other than those stated in the inclusion criteria, conference abstracts, editorials, books, dissertations, and other reviews.

Information Sources and Search Strategy

We performed a comprehensive search of four electronic databases (PubMed, Scopus, Web of Science, and Cochrane CENTRAL) from inception until Dec 15, 2023, using the following query, accustomed to each database: “((((Atrial OR Auricular OR atrium OR Nonvalvular OR "Non-valvular") AND (Fibrillation OR Fibrillations OR flutter OR flutters)) OR NVAF OR AFib OR AF) AND (((Atrial OR Auricular OR atrium OR left) AND (Appendage OR Appendages) AND (closure OR occlusion OR occlusions)) OR LAAC OR LAA) AND ((("non‐ vitamin K" OR direct OR novel OR new) AND (oral OR acting) AND (Anticoagulants OR Anticoagulant OR "Factor Xa" OR "Factor 10a")) OR (DOACs OR DOAC OR NOAC OR NOACs)))”. Furthermore, a manual search was conducted to ensure the inclusion of potentially missing relevant articles.

Selection Process

Endnote version X8 (Clarivate Analytics, PA) was used to remove the duplicates, and then the final retrieved references were screened in two phases: the first phase was to screen titles and abstracts of the retrieved articles independently by two authors (GRRN and LBDM). Next, the full-text articles were assessed for final inclusion in both qualitative and quantitative syntheses independently by two authors (GRRN and LBDM). Any discrepancies during screening were resolved by consensus or by consulting with third and fourth reviewers (RHS and APL). Another author (APL, a vascular surgeon from Sorocaba Hospital) was the most experienced researcher in our group, who was responsible for supervising the development of the article made by the members affiliated with Anhembi Morumbi University).

Data Collection Process and Data Items

Data were extracted using a uniform data extraction sheet. The extracted data included (1) general characteristics summary of the included studies, (2) baseline characteristics of the population of the included studies, (3) quality assessment of the included studies, and (4) appropriate measures of the targeted outcome.

Assessing the Risk of Bias in Individual Studies

Quality assessment of the included studies was performed independently and cross-checked by two authors (GRRN and LBDM). The Newcastle-Ottawa Scale (NOS) was used to evaluate the risk of bias in the included observational studies [10]. Selection (0-4 stars), comparability (0-2 stars), and outcome assessment (0-3 stars) are the three main domains of the NOS tool. Scores (7-9) indicated high quality, scores (4-6) demonstrated moderate quality, and scores (0-3) indicated low quality. In contrast, the Revised Cochrane Risk of Bias tool (ROB 2) was used to assess the quality of the included RCTs [11].

Synthesis Methods

All dichotomous outcomes were presented as event and total in addition to the odds ratio (OR) and their 95% confidence interval (CI) between the LAAC and DOACs groups. Continuous outcomes were presented as mean and standard deviation (SD) in addition to mean difference (MD) and their 95% CI. They were then pooled using Review Manager (RevMan) software (version 5.3; The Cochrane Collaboration, Copenhagen, Denmark). Both fixed and random effects models were used. However, the Mantel-Haenszel random effects model was referenced only in the case of statistically significant heterogeneity. Heterogeneity was estimated using the chi-square test (Cochrane Q test) (p < 0.1 implies significant heterogeneity) and quantified using the I2 statistic (I2 > 50% indicates significant heterogeneity).

Results

Literature Search Results

**Figure 1:**
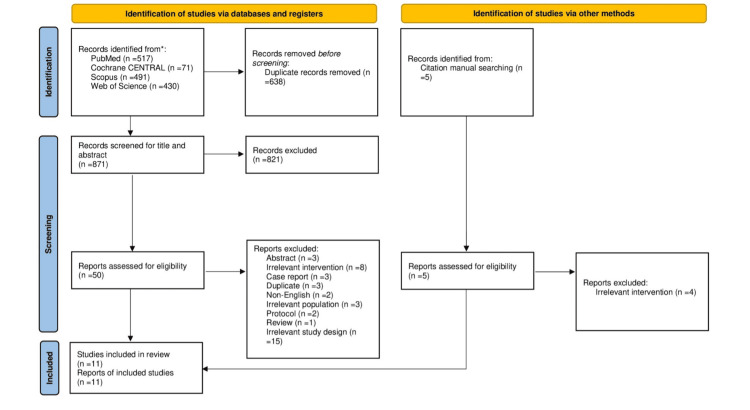
PRISMA flow chart of the study selection process. PRISMA = Preferred Reporting Items for Systematic Reviews and Meta-Analyses Source: Ref. [8]

Our literature search process retrieved 1,509 records. The results for each database were as follows: PubMed (517), Web of Science (430), Scopus (491), and Cochrane CENTRAL of controlled trials (71). After duplicate removal, 871 articles were processed for title and abstract screening. Fifty articles were eligible for full-text screening. Of these, 10 were included in the meta-analysis. The references of the included studies were manually searched, resulting in five potential records. After screening the manually retrieved records, only one article was included, resulting in 11 articles that were finally included in both the qualitative and quantitative analysis. A PRISMA flow diagram (Figure 1) of the study selection process is shown below. Ten observational studies [12-21] and one RCT [22] were included.

Characteristics of the Included Studies

**Figure 2:**
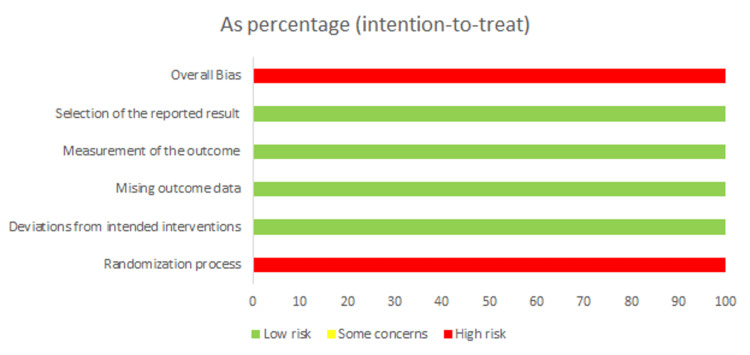
The RoB2 tool for quality assessment of Osmancik et al. 2022/PRAGUE-17 trial. Source: Ref. [9]

Eleven studies were included in the meta-analysis, with 571,631 patients with AF. In all studies, patients were allocated to either the LAAC (n = 12,224 patients) or DOACs (n = 559,407) group. A summary of the general characteristics of the included studies is presented in Table 1. The baseline characteristics of the included studies are presented in Table 2. Overall, the risk of bias in the included observational studies was of high quality according to NOS (Table 3). Additionally, the risk of bias in the included RCT was high according to the Cochrane RoB2 tool (Figure 2).

**Table 1:** A summary of the general characteristics of the included studies. Standard deviation = SD; years = yrs; DOAC = direct oral anticoagulation; LAAC = left atrial appendage closure; TIA = transient ischemic attack; SE = systemic embolism; AMI = acute myocardial infarction; ISTH = International Society of Thrombosis and Hemostasis; N/A = not applicable

Study ID	Study design	Country	Age, yrs, mean (SD)	Type of left atrial appendage device	Name of DOAC, n (%)	Follow-up duration, mean (SD)	Primary endpoint	Definition of primary composites
Caneiro-Queija, 2022 [14]	Propensity-score matched cohort study	Spain and Canada	85.7 ± 3.1 yrs	N/A	N/A	2 ± 0.43 years	Composite of death, major bleeding, and stroke	Composite of death, major bleeding, and stroke
Ding et al., 2022 [19]	Propensity-score matched cohort study	Global across 11 countries	69.55 ± 11.74 yrs	N/A	Apixaban: 340 (51.4%) Dabigatran: 99 (15.0%) Edoxaban: 10 (1.5%) Rivaroxaban: 230 (34.8%)	2 years	All-cause mortality, composite thrombotic and thromboembolic events, ischemic stroke or TIA, venous thromboembolism, and intracranial hemorrhage.	Composite thrombotic and thromboembolic events
Melillo et al., 2023 [16]	Single-center, observational prospective study	Italy	74.55 ± 6.97 yrs	Watchman, Amplatzer Cardiac Plug, Amplatzer Amulet	Dabigatran, apixaban, rivaroxaban	4.8 ± 3.06 years	Major bleeding, thromboembolic events: ischemic stroke, TIA, SE, AMI	Composite endpoint of thromboembolic events (ischemic stroke, TIA, AMI, SE) and ISTH major bleeding
Ng et al., 2023 [17]	Cohort study	Hong Kong	75.9 ± 8.7 yrs	N/A	Dabigatran, rivaroxaban, apixaban, edoxaban	2.88 ± 1.899 years	Composite of all-cause mortality, ischemic stroke, and major bleeding	Composite of all-cause mortality, ischemic stroke, and major bleeding
Nielsen-Kudsk et al., 2021 [12]	Propensity-score matched cohort study	Denmark	75.1 years yrs	Amplatzer Amulet device	N/A	2 years	Composite of ischemic stroke, systemic embolism, major bleeding, or all-cause mortality	Ischemic stroke, major bleeding, mortality
Osmancik et al., 2022 [22]	Prospective, open-label, RCT	Czech Republic	73.3 yrs	Amplatzer Amulet: 111 (61.3%) Watchman: 65 (35.9%) Watchman-FLX: 5 (2.8%)	Apixaban: 192 (95.5%) Dabigatran: 8 (4%) Rivaroxaban: 1 (0.5%)	4 years	Composite of characteristics of stroke, TIA, systemic embolism, clinically significant bleeding, cardiovascular death, or significant peri-procedural or device-related complications.	All-cause stroke, systemic embolism, or cardiovascular death
Paiva et al., 2021 [20]	Single-center, observational cohort study	Portugal	76.6 ± 8.39 yrs	N/A	N/A	1.08 ± 0.6 years	Composite endpoint of all-cause mortality, non-fatal stroke, and major bleeding	Composite endpoint of death, stroke, and major bleeding
Tiosano et al., 2023 [15]	Prospective cohort studies	Israel	77.3 ± 10.4 yrs	Amplatzer Amulet or Watchman	Rivaroxaban n = 1023, Apixaban n = 1164, and Dabigatran n = 951	1 year	All-cause mortality at one year	N/A
Turagam et al., 2023 [13]	Retrospective cohort study	US and Europe	74.3 ± 12.5 yrs	Watchman and Watchman-FLX devices, Amplatzer Cardiac Plug, and Amulet devices	Apixaban, rivaroxaban, dabigatran, or edoxaban	N/A	The incidence of disabling/fatal ischemic stroke at discharge and 3 months	N/A
Noseworthy et al., 2022 [21]	Cohort study	The U.S.	76.4 ± 7.6 yrs	N/A	Apixaban, dabigatran, edoxaban, or rivaroxaban	1.5 ± 1.0 years	Composite endpoint of ischemic stroke or systemic embolism, major bleeding, and all-cause mortality	Ischemic stroke or systemic embolism, major bleeding, and all-cause mortality
Deng et al., 2023 [18]	Prospective cohort study	The U.S.	≥ 18 yrs	Watchman 2.5 or Watchman FLX devices	Apixaban, rivaroxaban, dabigatran, or edoxaban	1.9 ± 1.56 years	severity of fall-related injuries	N/A

**Table 2:** Baseline characteristics of the included studies. CAD = coronary artery disease; CHF = congestive heart failure; DM = diabetes mellitus; DOAC = direct oral anticoagulation; HTN = hypertension; LAAC = left atrial appendage closure; N/A = not applicable

Baseline sample characteristics	Intervention	Summary statistics	Caneiro-Queija et al. 2022 [14]	Ding et al., 2022 [19]	Melillo et al., 2023 [16]	Ng et al., 2023 [17]	Nielsen-Kudsk et al., 2021 [12]	Osmancik et al., 2022 [22]	Paiva et al., 2021 [20]	Tiosano et al., 2023 [15]	Turagam et al., 2023 [13]	Noseworthy et al., 2022 [21]	Deng et al., 2023 [19]
No.	LAAC	Number	58	661	96	874	1071	201	91	114	91	8397	570
	DOAC	Number	58	661	96	1476	1184	201	149	342	91	554453	696
Age (year), mean (SD)	LAAC	Mean	85.6	69.9	73.8	75.5	75.1	73.4	74.7	77.9	76.2	75.8	78.9
		SD	2.5	10.8	7.1	8.2	8.5	6.7	8.7	7.44	14.7	7.2	8.1
	DOAC	Mean	85.8	69.2	75.3	76.2	75.1	73.2	77.8	77.1	76	75.8	79.1
		SD	3.7	12.6	6.8	9	10.5	7.2	8	11.2	10.7	7.2	10
Male, n (%)	LAAC	Number	32	428	54	534	687	134	59	70	48	4601	327
		%	55.2	64.8	56.3	61.1	64.2	66.7	64.8	61.4	52.7	54.8	57.4
	DOAC	Number	31	444	78	815	727	130	69	202	47	303840	365
		%	53.4	67.2	81.3	55.2	61.4	61.4	46.3	59.1	51.6	54.8	52.5
DM, n (%)	LAAC	Number	17	225	24	263	333	73	32	50	23	4408	N/A
		%	29.3	34.0	25.0	30.1	31.1	36.3	35.2	43.9	25.2	52.5	N/A
	DOAC	Number	19	207	23	447	424	90	36	137	24	291088	N/A
		%	32.8	31.3	24.0	30.3	35.8	44.8	24.2	40.1	26.4	52.5	N/A
HTN, n (%)	LAAC	Number	51	462	80	586	896	186	79	98	79	8246	N/A
		%	87.9	69.9	83.3	67	83.7	92.5	86.8	86	86.8	98.2	N/A
	DOAC	Number	47	455	90	1004	1023	186	123	279	75	53473	N/A
		%	81	68.8	93.8	68	86.5	85.5	82.6	81.6	82.4	98.2	N/A
CAD, n (%)	LAAC	Number	8	369	N/A	421	346	N/A	13	N/A	N/A	6407	N/A
		%	13.8	55.8	N/A	48.2	32.3	N/A	14.3	N/A	N/A	76.3	N/A
	DOAC	Number	14	389	N/A	639	402	N/A	22	N/A	N/A	423048	N/A
		%	24.1	58.9	N/A	43.3	33.9	N/A	14.8	N/A	N/A	76.3	N/A
Heart Failure, n (%)	LAAC	Number	20	236	N/A	235	178	88	42	44	N/A	4778	N/A
		%	34.5	35.7	N/A	26.9	16.6	43.8	46.2	38.6	N/A	56.9	N/A
	DOAC	Number	22	242	N/A	449	223	90	34	124	N/A	315484	N/A
		%	37.9	36.6	N/A	30.4	18.9	44.8	22.8	36.3	N/A	56.9	N/A
Previous stroke, n (%)	LAAC	Number	14	45	34	325	333	66	31	49	36	2821	N/A
		%	24.1	6.8	35.4	37.2	31.1	32.8	34.1	43	39.6	33.6	N/A
	DOAC	Number	16	46	28	557	376	63	46	127	30	186296	N/A
		%	27.6	7.0	29.2	37.7	31.8	31.3	30.9	37.1	33.0	33.6	N/A
CHA₂DS₂-VASc score, mean (SD)	LAAC	Mean	4.7	N/A	4.3	4.4	4.2	4.7	4.3	4.17	5	143	N/A
		SD	1.1	N/A	1.5	1.6	1.6	1.5	1.4	1.29	1.5	1.7	N/A
	DOAC	Mean	4.7	N/A	4.3	4.5	4.3	4.7	5.3	3.96	5	9980	N/A
		SD	1.5	N/A	1.5	1.7	1.7	1.5	1.3	1.57	1.5	1.8	N/A
HASBLED score, mean (SD)	LAAC	Mean	3.2	N/A	3.5	4.6	3.3	3.1	3	4.14	3	N/A	N/A
		SD	0.7	N/A	0.7	1.4	1	1.9	0.9	1.04	1.5	N/A	N/A
	DOAC	Mean	3.2	N/A	3.5	4.6	3.4	3	4	3.95	3	N/A	N/A
		SD	1	N/A	0.6	1.4	1.2	1.9	0.7	1.35	1.5	N/A	N/A

**Table 3:** The Modified Newcastle-Ottawa Scale for quality assessment of the included observational studies. * represents a "star", meaning the article got a "checkmark" for that criteria (i.e., it fulfilled the criteria); ** means highly comparable and hence receives two; --- may be read as "blank" or "N/A".

Study ID	Selection				Comparability	Outcome			Total	Quality
	Representativeness of the exposed cohort	Selection of the non-exposed cohort	Ascertainment of exposure	Demonstration that outcome of interest was not present at the start of the study	Comparability of cohorts based on the design or analysis	Assessment of outcome	Was follow-up long enough for outcomes to occur?	Adequacy of follow-up of cohorts	No. of points	Overall quality
Caneiro-Queija et al., 2022 [14]	*	*	*	*	**	*	*	*	9	High
Ding et al., 2022 [19]	*	*	*	*	**	*	*	*	9	High
Melillo et al., 2023 [16]	*	*	*	*	**	*	*	*	9	High
Ng et al., 2023 [17]	*	*	*	*	**	*	*	*	9	High
Nielsen-Kudsk et al., 2021 [12]	*	---	*	*	**	*	*	*	8	High
Paiva et al., 2021 [20]	*	*	*	*	**	*	*	---	8	High
Tiosano et al., 2023 [15]	*	*	*	*	**	*	*	---	8	High
Turagam et al., 2023 [13]	*	---	*	*	**	*	*	---	7	High
Noseworthy et al., 2022 [21]	*	*	*	*	**	*	*	*	9	High

Meta-Analysis

**Figure 3:**
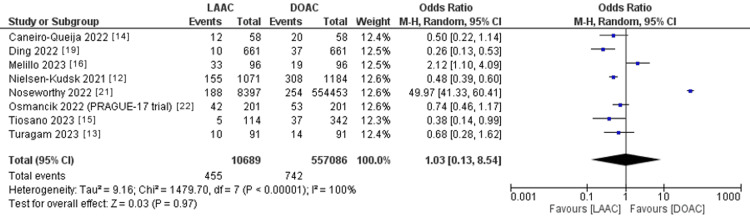
Forest plot of the composite outcome of stroke, systemic embolism, and cardiovascular death. I^2 ^= heterogeneity; 95% CI = CI: confidence interval; LAAC = left atrial appendage (LAA) closure; DOAC = direct oral anticoagulants; M-H, Random = Mantel-Haenszel random-effects model Sources: Refs. [12-16,19,21,22]

The pooled OR of the composite outcome (Figure 3) did not statistically favor either of the two study groups (OR: 1.45; 95% CI: 0.81-11.55; p = 0.72). The pooled studies were heterogeneous (p < 0.001; I^2^ = 100%). 

**Figure 4:**
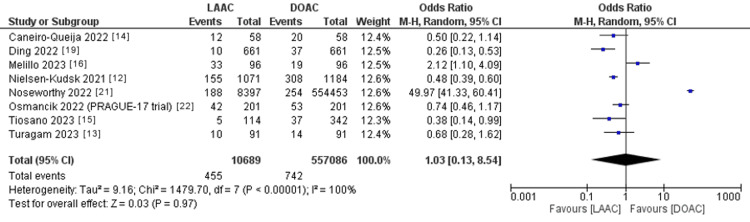
Forest plot of all-cause mortality. I^2^ = heterogeneity; 95% CI = CI: confidence interval; LAAC = left atrial appendage (LAA) closure; DOAC = direct oral anticoagulants; M-H, Random = Mantel-Haenszel random-effects model Sources: Refs. [12-16,19,21,22]

The pooled OR of all-cause mortality (Figure 4) did not statistically favor either of the two study groups (OR: 1.03; 95% CI: 0.13-8.54; p = 0.97). The pooled studies were heterogeneous (p < 0.001; I^2^ = 100%).

**Figure 5:**
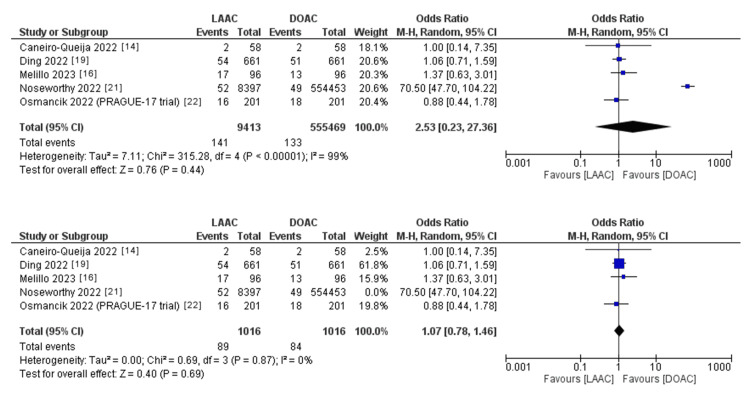
Forest plot of ischemic stroke and thromboembolic events before and after leave-one-out analysis for Noseworthy et al. I^2 ^= heterogeneity; 95% CI = CI: confidence interval; LAAC = left atrial appendage (LAA) closure; DOAC = direct oral anticoagulants; M-H, Random = Mantel-Haenszel random-effects model Sources: Refs. [14,16,19,21,22]

The pooled OR of ischemic stroke and thromboembolic events (Figure 5) did not statistically favor either of the two study groups (OR: 2.53; 95% CI: 0.23-27.36; p = 0.44). The pooled studies were not homogenous (p < 0.001; I^2^ = 99%). However, when the study by Noseworthy et al. was removed through leave-one-out analysis, the pooled studies were homogenous (p = 0.87; I^2^ = 0%).

**Figure 6:**
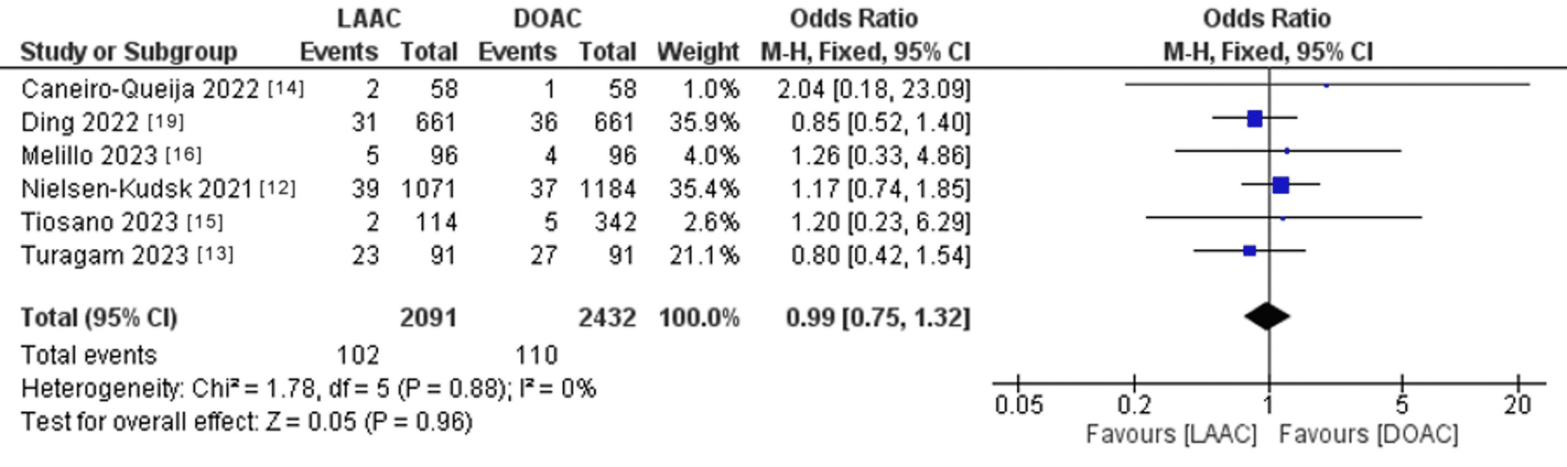
Forest plot of ischemic stroke. I^2^ = heterogeneity; 95% CI = CI: confidence interval; LAAC = left atrial appendage (LAA) closure; DOAC = direct oral anticoagulants; M-H, Random = Mantel-Haenszel random-effects model Sources: Refs. [12-16,19]

The pooled OR of ischemic stroke (Figure 6) did not statistically favor either of the two study groups (OR: 0.99; 95% CI: 0.75-1.32; p = 0.96). The pooled studies were homogenous (p = 0.88; I^2^ = 0%).

**Figure 7:**
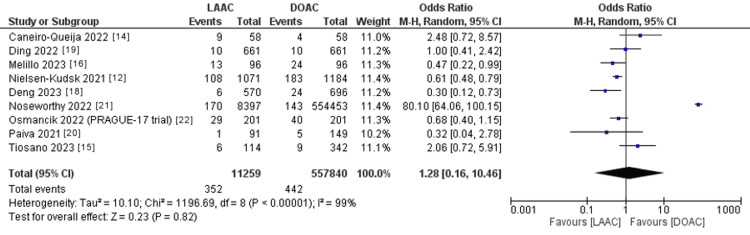
Forest plot of major bleeding. I^2^ = heterogeneity; 95% CI = CI: confidence interval; LAAC = left atrial appendage (LAA) closure; DOAC = direct oral anticoagulants; M-H, Random = Mantel-Haenszel random-effects model Sources: Refs. [12,14-16,18-22]

The pooled OR of major bleeding (Figure 7) did not statistically favor either of the two study groups (OR: 1.28; 95% CI: 0.16-10.46; p = 0.82). The pooled studies were heterogeneous (p < 0.001; I^2^ = 99%).

**Figure 8:**
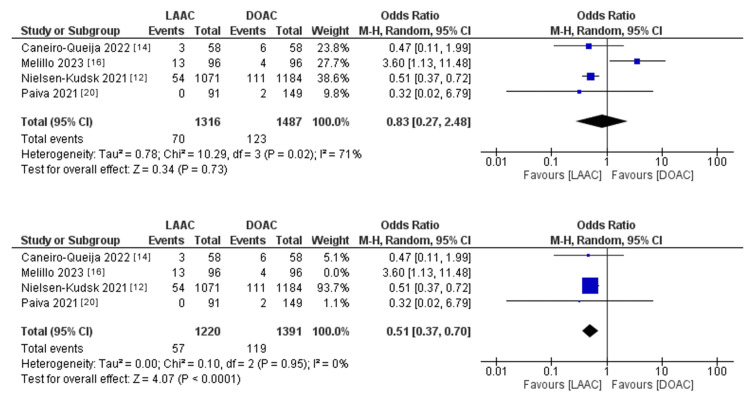
Forest plot of cardiovascular mortality events before and after leave-one-out analysis for Melillo et al. I^2^ = heterogeneity; 95% CI = CI: confidence interval; LAAC = left atrial appendage (LAA) closure; DOAC = direct oral anticoagulants; M-H, Random = Mantel-Haenszel random-effects model Sources: Refs. [12,14,16,20]

The pooled OR of cardiovascular mortality (Figure 8) did not statistically favor either of the two study groups (OR: 0.83; 95% CI: 0.27-2.48; p = 0.73). The pooled studies were heterogeneous (p = 0.02; I^2^ = 71%). However, when Melillo et al.'s study was removed through leave-one-out analysis, the pooled studies were homogenous (p = 0.95; I^2^ = 0%).

**Figure 9:**
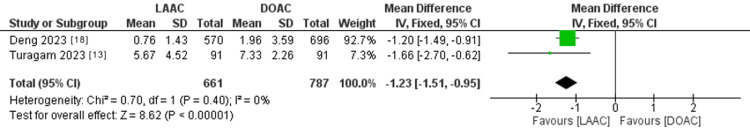
Forest plot of length of hospital stay duration. I^2^ = heterogeneity; 95% CI = CI: confidence interval; LAAC = left atrial appendage (LAA) closure; DOAC = direct oral anticoagulants; M-H, Random = Mantel-Haenszel random-effects model Sources: Refs. [13,18]

The pooled MD of length of hospital stay (Figure 9) was statistically lower in the LAAC group than in the DOAC group (MD: -1.23; 95% CI: -1.51 to -0.95; p < 0.001). The pooled studies were homogenous (p = 0.40; I^2^ = 0%).

Discussion

AF is a significant global public health problem owing to its association with coronary heart disease, stroke, and mortality, leading to further strain in healthcare systems [2]. Asymptomatic patients with AF may present with stroke as the first AF symptom, necessitating vital care for this category of patients [4]. Therefore, several treatment options have been developed to prevent stroke and thromboembolic events in patients with AF, including traditional warfarin therapy, DOACs, and the recent LAAC treatment approach. Multiple systematic reviews and meta-analyses have compared the safety and efficacy of LAAC and DOACs in patients with AF. However, they had a smaller sample size and lacked some important outcomes, such as hospital stay duration [4-6].

This updated systematic review and meta-analysis of 571,631 patients compared the safety and efficacy of LAAC and DOACs in patients with AF. The main findings of our analysis were as follows: (1) the potential advantage of LAAC over DOACs in terms of hospital stay duration and (2) no statistically significant difference between LAAC and DOACs in terms of composite outcomes of stroke, systemic embolism, cardiovascular death, all-cause mortality, ischemic stroke and thromboembolic events, ischemic stroke alone, major bleeding, and cardiovascular mortality.

The apparent advantage of LAAC over DOACs in terms of hospital stay duration can be explained by the potential decrease in bleeding risk and the need for long-term anticoagulation therapy. This finding is of paramount importance because the literature lacks information regarding hospital stay duration in patients with AF receiving either LAAC or DOACs. This shorter length of hospital stay may positively impact healthcare systems in terms of cost and expenses [23].

Furthermore, the statistically insignificant difference between LAAC and DOACs in terms of the composite outcome of stroke, systemic embolism, and cardiovascular death was inconsistent with that of Jiang et al., who found that the composite outcome was lower with LAAC [5]. This can be explained by the larger sample size in our study. Similarly, Al-Abacha et al. found a lower risk of composite outcomes in the LAAC group [4]. The high heterogeneity between the pooled studies can be explained by the different definitions of the composite outcome and the different follow-up durations among them. The similar rates of ischemic stroke and thromboembolic events between the LAAC and DOACs groups were consistent with those reported by Jiang et al. and Chen et al. [5,6]. In addition, the high heterogeneity was resolved through the leave-one-out analysis of Noseworthy et al. [21], which might be explained by the relatively small follow-up duration (1.5 ± 1.0 years), with the largest sample size in both arms among all the included studies.

The all-cause mortality rate was similar between the LAAC and DOAC groups, which agrees with the results of Chen et al. [6]. In contrast, Jiang et al. and Al-Abacha et al. demonstrated a lower rate with LAAC [4,5]. Similarly, Chen et al. [6] reported similar cardiovascular mortality rates between the two arms. However, Jiang et al. and Al-Abacha et al. demonstrated a lower rate with LAAC [4,5]. Moreover, high heterogeneity was resolved through the leave-one-out analysis of Melillo et al. [16], which might be explained by the long follow-up duration (4.8 ± 3.06 years). The decrease in the rate of all-cause and cardiovascular mortality events is potentially multifactorial in origin and might be explained by the decreased incidence of hemorrhagic stroke events [4].

Although our study has some strengths, including a large number of included patients while reporting a novel potential outcome (length of hospital stay), it also has some limitations: (1) our pooled analysis was performed on the aggregate data from all the studies, which were not individual-level data; (2) multiple devices and DOACs were used in the included studies with the inability for a head-to-head comparison; (3) the high heterogeneity among most of the reported meta-analyzed outcomes hinders the generalization of our findings; and (4) the meta-analysis involved the combined analysis of one RCT and 10 observational studies because of the lack of RCTs in this comparison. Given that the certainty of the evidence from observational studies is low in general, our findings are not entirely conclusive and should be interpreted with caution.

## Conclusions

Our pooled analysis revealed no statistically significant difference between LAAC and DOACs in terms of various clinical outcomes, including stroke, systemic embolism, cardiovascular death, all-cause mortality, ischemic stroke, thromboembolic events, ischemic stroke alone, major bleeding, and cardiovascular mortality. Despite this, the analysis highlighted a potential advantage of LAAC over DOACs in terms of reducing the duration of hospital stay, suggesting a possible novel benefit in this specific aspect.

Our findings warrant the necessity for RCTs with larger sample sizes and longer unified follow-up durations. Additionally, the implementation of a standardized definition for composite outcomes is crucial to address the ongoing debate concerning the safety and efficacy of LAAC compared to DOACs. Such rigor in future studies will be essential in providing more definitive conclusions.
